# Medical Note Quality Audit at the Vascular Surgical Unit Compared With the British Medical Journal (BMJ) Guidelines

**DOI:** 10.7759/cureus.50110

**Published:** 2023-12-07

**Authors:** Nicolette Busuttil, Kevin Cassar, Gordon Caruana Dingli, Matthew Joe Grima

**Affiliations:** 1 Surgery, Mater Dei Hospital, Msida, MLT; 2 Vascular Surgery, Mater Dei Hospital, Msida, MLT; 3 Vascular Surgery, University of Malta, Msida, MLT; 4 Breast Surgery, Mater Dei Hospital, Msida, MLT; 5 General Surgery, University of Malta, Msida, MLT; 6 Vascular Surgery, Uppsala University Hospital, Uppsala, SWE

**Keywords:** history and physical note, electronic health records, artificial intelligence in medical note writing, medical note writing, cardiothoracic & vascular surgery

## Abstract

Effective medical notes ensure comprehensive documentation in healthcare. This study evaluates medical note quality in the vascular unit at Mater Dei Hospital using *British Medical Journal *(*BMJ*) guidelines. Two cycles examine 17 parameters pre- and post-intervention, revealing notable, significant enhancements in patient identifiers, clinical summaries, examination, and planning. Future prospects involve digitizing note-taking, utilizing artificial intelligence (AI) for data organization, and simplifying entry methods. Implementation of electronic solutions is encouraged for improved accuracy, efficiency, and continuity of patient care.

## Introduction

Malta, an island in the Mediterranean Sea with approximately 535,000 inhabitants, is served by a sole primary acute hospital, Mater Dei Hospital. This audit was conducted within the Vascular Surgery Unit within Mater Dei Hospital. The primary objective was to assess how clinical notes align with the standards and recommendations set by the *British Medical Journal* (*BMJ*) guidelines [1]. The audit will play a key role in identifying the existing gaps in medical notes in the healthcare facility and will play a central role in the future improvement of medical note-taking among doctors in the vascular surgical department and potentially other departments for the purpose of improving the overall health quality, handover, and continuity of care for patients [2]. Through this assessment, the audit aims to ascertain whether any of the implemented interventions have contributed to improvements in the quality of medical notes produced by the unit.

Medical notes are pivotal for ensuring patient safety, continuity of care, and accurate communication among healthcare professionals and also are important medico-legal documents. 

## Materials and methods

Seventeen parameters for medical notes were chosen carefully using the *BMJ *guidelines, which offer peer-reviewed, trusted guidelines, which the audit followed. They ensure clear and detailed patient records which are key for safety and good communication. The parameters include patient ID card number, patient name, the date and time when the medical notes were written, the ward in which the medical notes were written, names/surnames of senior clinicians/surgical clinicians present during the ward round, broad summary of the situation when the notes were written (for example, day 1 post-op or day 1 post cellulitis). In addition, the audit will examine whether the notes consist of both subjective and objective comments on the health conditions of patients (for example, ‘patient seen mobilizing'); other elements that will be audited will include examination, parameters, impression, plan; signature, designation, and contact information/registration number of the person writing the medical notes, and legibility.

Ethical approval was obtained before data collection. 

First cycle of data collection

The first cycle of this medical audit comprised 25 consecutive patients admitted to the vascular unit wards at the Mater Dei Hospital. The patients were admitted to two vascular wards at the Mater Dei Hospital. 

In the first cycle, it was ensured that all the patients had been seen by a vascular surgeon firm +/- consultations with other surgical/medical specialties. The collection of health information from these patients was done retrospectively from all patients who had been discharged during a one-week period. The data was collected over 1 week per cycle, and there was a 2-month difference between cycles one and two.

All the information about the medical entries was then gathered in a Microsoft Excel worksheet (Microsoft Corporation, Redmond, USA) using a checklist that examined 17 parameters cited in Table 1. This audit included all surgical patients regardless of the type of procedure that had been applied in the course of their treatment. Parameters, including notes written by allied healthcare professionals, were excluded.

**Table 1:** Parameters for data collection

Parameter	Description
1.	ID Card Number
2.	Name
3.	Surname
4.	Date
5.	Time
6.	Location in Hospital
7.	Name/Surname of Most Senior Clinician During Ward Round (WR)
8.	Single Summary of Broad Clinical Situation
9.	Subjective and Objective Comments on Patient's Condition
10.	Examination
11.	Parameters
12.	Impression
13.	Plan
14.	Signature of the Person Writing the Note
15	Designation of the Person Writing the Note
16.	Contact Number of the Person Writing the Note
17.	Legibility

In order to eliminate any form of performance bias during data collection, the ward-round firms were not informed of the plan to have the audit prior to the collection of the information. Random selection of the data collection week was done using an online random picker. The parameters being measured were objective.

The results were presented at the vascular surgery consultants’ meeting following the completion of data collection and feedback was taken into consideration for the next cycle.

Second cycle of data collection

The second cycle comprised 22 patients, with three patients not being vascular patients, coming from the two vascular wards - using the same settings as earlier. 

**Figure 1:**
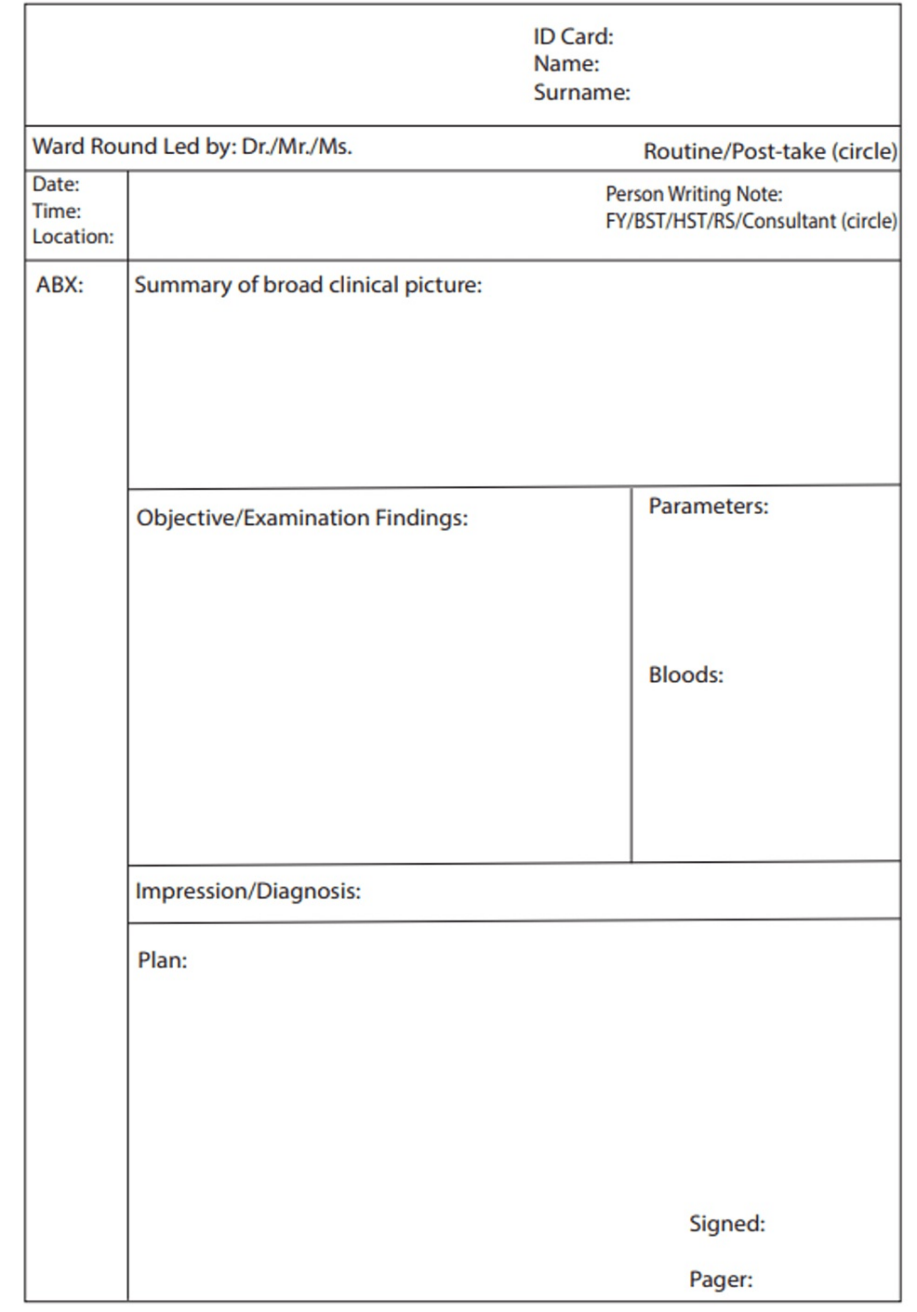
Proforma for 'Ideal Note Writing' Developed by Dr. Nicolette Busuttil, the figure is a structured compilation based on parameters derived from the *British Medical Journal *guidelines*.*

Clinical members of the vascular surgery unit, including consultants and junior doctors, were made aware of the issues. A proforma for ‘ideal note writing’ as per the *BMJ *guidelines was sent via email (Figure 1). Stickers that had 'Name, Surname and ID-Card' were printed and affixed to the top of the papers used for medical note-taking.

Members of the ward round teams comprising foundation doctors, basic specialist trainees, higher specialist trainees, and resident specialists were also verbally informed, with weekly reminders. The data was collected blindly again without the members knowing when the data collection was going to happen.

## Results

Table 2 and Table 3 present the data collected in the first and second cycles of the study, and they present the number of entries for each of the audited 17 parameters as a percentage of the total ward round entries (265 and 264 entries, respectively). This section entails a comprehensive analysis of the contrasting percentage of records for each parameter before and after the intervention. The study encompassed patients admitted to the vascular unit wards of the Mater Dei Hospital, with data collected during two distinct cycles.

**Table 2:** Percentage of each audited parameter present in 265 ward round entries (cycle one)

Parameter	Percentage of each audited parameter present in 265 ward round entries (cycle one)
ID Card Number	89.0
Name	93.5
Surname	93.1
Date	93.9
Time	65.5
Location in Hospital	56.8
Name/Surname of Most Senior Clinician During Ward Round (WR)	63.6
Single Summary of Broad Clinical Situation	68.2
Subjective and Objective Comments on Patient's Condition	76.1
Examination	64.0
Parameters	59.5
Impression	13.6
Plan	100
Signature of the Person Writing the Note	92.8
Designation of the Person Writing the Note	22.7
Contact Number of the Person Writing the Note	1.9
Legibility	95.1

**Table 3:** Percentage of each audited parameter present in 264 ward round entries (cycle two)

Parameter	Percentage of each audited parameter present in 264 ward round entries (cycle two)
ID Card Number	96.8
Name	94.5
Surname	94.5
Date	90.9
Time	88.1
Location in Hospital	76.3
Name/Surname of Most Senior Clinician During Ward Round (WR)	87.4
Single Summary of Broad Clinical Situation	81.4
Subjective and Objective Comments on Patient's Condition	80.2
Examination	84.2
Parameters	77.9
Impression	13.4
Plan	99.6
Signature of the Person Writing the Note	93.3
Designation of the Person Writing the Note	40.3
Contact Number of the Person Writing the Note	8.3
Legibility	99.6

A direct contrast of the percentage number of records for each parameter before and after the intervention revealed notable disparities and improvements across various aspects of medical note-writing.

The percentage records for 'ID Card Number', 'Name', and 'Surname' exhibited a significant increase after the intervention, aggregating 97%, 94%, and 94% for each parameter (from 89%, 93%, and 93%, respectively). This indicates a substantial improvement in the thorough inclusion of these fundamental patient identifiers.

The percentage of records for 'Date' slightly decreased to 91% (from 94%). 'Time' also experienced an increase to 88% (from 66%). The percentage for 'Location in Hospital' increased to 76% (from 57%). The percentage for the 'Name/Surname of Most Senior Clinician During Ward Round (WR)' increased from 63% to 87%. The percentage for 'Single Summary of Broad Clinical Situation' increased to 81% from 68%. The percentage for 'Subjective and Objective Comments on Patient's Condition' increased to 80% from 76%. The percentage for 'Examination' increased to 84% (up from 64%), signifying a more thorough examination notation. Similarly, 'Parameters' exhibited a percentage increase to 78% (up from 59%), reflecting enhanced documentation of medical parameters. The percentage for 'Impression' slightly decreased to 13% (from 14%). The 'Plan' parameter showed an increase to 100% (from 99.6%). The percentage of ' Signature of the Person Writing the Note' slightly increased to 93% (from 92%). The percentage for 'Designation of the Person Writing the Note' also increased to 40% (from 23%). There was an increase in the percentage for the 'Contact Number of the Person Writing the Note' to 8% (from 2%). The 'Legibility' parameter exhibited a noteworthy increase to 100% (up from 95%), emphasizing a heightened emphasis on readability and clarity in medical notes.

**Figure 2:**
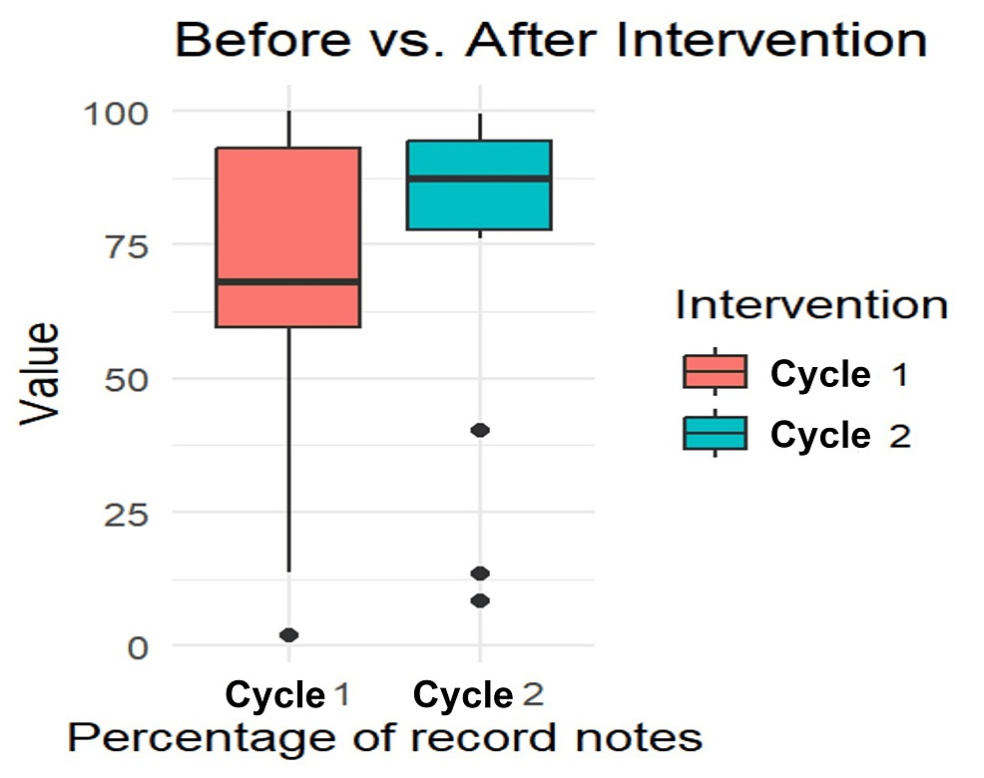
Paired t-test t (16) = -4.0987, p < 0.001

A paired t-test was conducted to examine significant differences for all parameters using R 4.0 programming software (R Foundation for Statistical Computing, Vienna, Austria). The paired t-test analysis, as seen in Figure 2, revealed a significant difference between the percentage of medical notes recorded before and after the intervention on all 17 parameters (t (16) = -4, p < 0.001). 

## Discussion

Importance of accurate medical records

Up-to-date medical records and accurate medical note-writing play a critical role in establishing the facts. Case notes have been shown to be the ‘best evidence’ in court cases, which usually occur many months or years after the patient has left the hospital. Missing data might have medical, financial, and medicolegal implications.

Innovations in medical records will enhance quality, but identifying and correcting deficiencies in established systems through auditing is also beneficial to identify any gaps in the quality provision. The standard of note-keeping can be improved with repeated audits and presentation of the results to the staff.

From the study's results, one can note that numerous parameters were not being documented well prior to the 2nd cycle. Several implementations were done, namely the proforma, the stickers applied at the top of the continuation sheet, reminders, and ‘pre-printed admission proformas to improve the quality of information recorded. Junior staff find them easier to use; they allow patients to be assessed faster and result in fewer tests being ordered. Filling in proformas requires less writing, and the use of tick boxes may prevent problems in retrieving information caused by poor writing [3]. Even though this might have been a good option, one has to also note the environmental and financial issues of printing a new proforma/sticker every time [4].

A poster, which illustrated the results and emphasised the importance of proper medical note-writing, was developed and shared with the entire vascular department. Included in the poster was the 'Ideal Note Writing Proforma,' as shown in Figure 1. This initiative, complemented by simple reminders and emails, played a significant role in enhancing overall outcomes.

Implementing a paperless system might have improved adherence to the guidelines, with less paper use and fewer human-induced errors [4,5]. Expanding this audit throughout the entire hospital and encompassing a larger population would be a compelling next step, allowing us to assess if any improvements can be identified on a broader scale.

One can note that educating the staff on medical note writing should be done hospital-wide - the improvements and implementations done would be beneficial to the whole hospital.

The audit was conducted in a 3-month period where the firms would have remained the same, however, one must note that in view of the ever-changing firms (every 3-6 months), this education and auditing must continue to occur to ensure high-quality note writing is present.

Interpretation of results

The contrasting percentage parameter records before and after the intervention highlight the substantial influence of interventions on medical note-writing practices. The significant improvement in patient identifiers, together with enhanced aspects such as contact details and legibility, attests to the success of interventions in promoting standardized, effective, and comprehensive documentation [6].

The percentage records for 'Date' slightly decreased to 91% (from 94%). 'Time' experienced an increase to 88% (from 66%). This might have been because more time was focused on the ‘ID card Number, Name and Surname’ stickers.

The percentage for 'Impression' slightly decreased to 13% (from 14%), reflecting no improvement in patient impressions. The Impression also remained low compared to other parameters as this is usually written in the 'Emergency Department' note.

There was an increase in the percentage for the 'Contact Number of the Person Writing the Note' to 8% (from 2%). However, it's essential to emphasize that this parameter, although showing improvement, is still relatively low compared to the other parameters. This observation can be attributed to the fact that in Malta (the study's environment), it might be often not considered a necessity to keep such a record when the information is readily available online. It is a common oversight by the people writing the note, but it should still be addressed. This observation underscores the need to raise awareness about the importance of including contact numbers, even when online information is readily available, in order to maintain comprehensive and reliable records.

It is important to note that this audit was conducted with a limited sample size, and as such, the data should be interpreted with caution.

Future of medical note-writing

Integrating medical note-writing education into undergraduate training presents numerous advantages, including the early cultivation of good habits, enhanced patient care, and the facilitation of adaptation to modern systems. By introducing note-writing practice at an early stage, students can steadily refine this skill, ensuring their proficiency in maintaining comprehensive patient records as they enter their professional careers. Discussions have been held with the Head of the Department of Surgery and the Head of the Department of the Medical School concerning this audit. There is a strong commitment to incorporating the valuable insights gained from this audit into both the medical school and the Foundation Programme curricula. [7]

The use of digital technologies would be the way forward to facilitate electronic medical note-taking and online access to medical notes. However, the path to integration is not without obstacles. From issues like suboptimal interface designs hindering user efficiency to incompatibilities between systems leading to gaps in patient care and data transfer, the challenges are multifaceted. Furthermore, resistance from some healthcare professionals rooted in their comfort of handwritten medical note-taking and concerns about privacy and security in the era of digital health records underscore multiple complex barriers. Acquiring, implementing, and maintaining new technologies and hardware will require a higher budget, further complicating the adoption of technology. Additionally, the need for robust training to ensure staff are well-acquainted with new systems is also essential. In the ensuing sections, we'll delve deeper into strategies to address and navigate these challenges [8-9].

Several studies have found that computer use in clinical settings has generally been accepted by patients, whether in general practice surgeries, outpatients, or examination rooms [10]. No effect on the patient-doctor relationship was noted [11-12].

A prospective randomized crossover study comparing handwritten paper notes to electronic notes on different patients by the same author confirmed that an electronic clinical documentation system improved note quality compared to handwritten documentation. Both the overall quality of documentation and the quality of free-text components of the documentation improved significantly [13].

Mater Dei Hospital is implementing the use of Patient Dashboard, which is currently used by multiple specialities in outpatient visits. Tools like Patient Dashboard, when integrated hospital-wide and out-of-hospital, like general practitioner (GP) clinics, can streamline outpatient visits, aiding in paperless medical note-writing. Implementation of ward medical note writing, with a proforma, check-list, and an area to fill in the clinical details (similar to the simplified version of the emergency department note proforma) would be revolutionary to create an online, paperless medical note-writing in Malta.

Digital technologies offer the advantage of easily incorporating elements such as checklists, mnemonics, algorithms, risk calculators, and local guidelines into a proforma, complete with automatic parameter linking. This level of integration and functionality surpasses what can be achieved with conventional handwritten notes [14].

In the healthcare setting, it is crucial to safeguard medical notes from interruptions, calls, and emergencies. This can be accomplished by utilising features such as real-time auto-save, cloud-based storage, offline mode with synchronization, automatic drafts (similar to email drafts), and backup systems. These methods can be implemented, allowing healthcare professionals to easily retrieve drafts and continue their work in case of interruptions. These measures guarantee the security and accessibility of vital patient data, thereby upholding the integrity of medical records [8-9].

Digital note-taking would also aid in discharge letters and the assimilation of the hospital stay. This idea would be beneficial if it is linked to several specialities hospital-wide, as well as GP centres, for continuity of care.

Making data entry as easy and quick as possible is essential if clinicians are to use electronic means to enter and share accurate patient records. One has to keep in mind that the easiest way to enter data might be speech, followed by handwriting and then typing. One could opt for handwritten - electronic devices such as tablets with a stylus - as one can note from the results that legibility was not a pressing issue [9,15].

Electronic medical note-writing consumes a significant amount of time and might compel physicians to gather an excessive amount of irrelevant data. Due to reasons like this, the quality of electronic health record (EHR)documentation has varied from below-par to unsatisfactory [16]. Artificial intelligence (AI), has the potential to alleviate the currently cumbersome process of collecting and structuring the components necessary for crafting a clinical note [16].

AI possesses the capacity to aid users in extracting pertinent information from various information systems (such as past electronic notes such as past medical history, bedside monitors, and imaging, abnormal laboratory results, and pharmacy systems). It can then organize this information in appropriate sections in the medical note. Furthermore, AI can scrutinize this assessment to construct a plan of action driven by data analysis [16].

Thoughtful utilization of autofill, particularly for data categories prone to repetition, could significantly reduce the need for excessive clicking and typing. AI could also assist in formatting and populating the note based on insights garnered from a user's past inputs and potentially those of the patient as well [17].

Parameters such as the history, examination, impression, and plan must still be inputted before they can be utilized for the purpose of note creation. However, potential solutions could encompass options like entering free-form text through voice recognition or keyboard input or utilizing natural language processing to transform unstructured text into organized data within the system. 'PhenoPad' is one of the technologies that are promising to change the landscape of clinical note-taking [17]. It is a hybrid tablet/keyboard device in combination with AI technologies. It captures both unstructured notes and standardized phenotypic information through diverse methods. These encompass speech recognition, natural language processing, handwriting interpretation, and more. The resulting data is seamlessly displayed on mobile devices for real-time clinician validation. Moreover, it can be automatically transmuted into digital formats compatible with integration into electronic health record systems [17].

These technologies will revolutionize healthcare by enhancing patient care and improving efficiency across various aspects of medical practice. Continuity of care will be greatly facilitated as electronic records allow healthcare providers to access a patient's complete medical history, enabling better-informed decisions and reducing the risk of errors. Handovers might be improved between healthcare teams, as electronic notes can be instantly shared and updated. Discharge letters will be generated more quickly, ensuring patients have comprehensive instructions for post-hospitalization care. Outpatient care will be improved through the merging of notes from different specialties, enabling comprehensive treatment plans. Furthermore, the shift towards paperless documentation reduces administrative burdens, lowers the risk of data loss, and contributes to a more eco-friendly healthcare system. Electronic medical note-writing is a vital tool in modern medicine, fostering better patient care and operational efficiency.

Limitations

While this study sought to provide valuable insights, it is important to acknowledge the limitations that influenced the audit.

This study was characterised by a relatively small sample size, which is a notable limitation. Future medical note audits could aim for a more comprehensive approach, potentially adopting a hospital-wide strategy for data collection. Despite the constraints imposed by the smaller sample size, the insights gained from this study are valuable and contribute to our understanding of medical note quality within the scope of the data available.

Another concern pertains to the subjectivity involved in assessing legibility - relying on a single data collector could introduce unintended biases in the assessment process.

The absence of historical data for comparative analysis was a notable constraint, as it hindered the ability to track changes over time.

Moreover, the study's focus solely on quantitative measures did not allow for a deeper exploration of the underlying reasons for the observed deficiencies in recording certain parameters within medical notes. The study did not delve into a qualitative analysis to uncover the nuances of why specific parameters were frequently omitted. To address these limitations and enhance the study's comprehensiveness, the implementation of the CRABEL score [18] may be utilised. The CRABEL score is a method for auditing the quality of medical record keeping. It provides a numerical score. The CRABEL score includes parameters like initial clerking, subsequent entries, consent, and discharge summary. The score's utility lies in its ability to offer a reproducible and straightforward objective assessment of the quality of medical record keeping. By expanding the auditing methodology to encompass a broader array of parameters and aspects, the study could gain a more holistic perspective. This approach would not only provide a more comprehensive evaluation but also offer opportunities for continuous improvement by identifying specific areas for intervention and refinement.

Considering the potential benefits of enhanced medical note quality, a pilot program introducing electronic medical note writing in the Vascular Surgical Unit or the hospital's entirety could be trialed. This way one could analyse the financial implications related to software adoption and training and the long-term effects in terms of data accuracy and accessibility. 

Medical note-writing is paramount for patient care and safety. Regular, detailed note-taking during ward rounds is often overlooked, yet it's critical to instill its importance early, during undergraduate training. Early emphasis ensures physicians cultivate good note-writing habits, benefiting patient care and facilitating adaptation to contemporary systems. Digital technology, like 'Patient Dashboard' and 'PhenoPad', promise enhanced electronic note-taking and data access. [17] However, challenges such as technology adoption barriers, resistance to change from traditionalists, and financial implications persist. Research indicates electronic documentation systems elevate note quality. Nonetheless, electronic note-taking may be time-consuming, sometimes leading to irrelevant data collection. AI's emergence, particularly in structuring and extracting relevant information, offers a potential solution, making documentation efficient and relevant. Moving forward, the focus should be on consistent improvements in medical documentation and understanding its profound effect on patient care quality and safety.

## Conclusions

In conclusion, this audit aimed to assess medical note quality at Mater Dei Hospital's vascular unit using *BMJ *guidelines. Two assessment cycles of 165 and 164 data entries evaluated interventions like proforma, note stickers, and reminders. Improvements were notable in patient identifiers, clinical details, examination notes, and legibility, enhancing note completeness. Despite favourable outcomes, suggestions for future improvements might include digital solutions for streamlined, accurate note-taking, like electronic records, AI-assisted documentation, and systems like PhenoPad.
